# The Main Factors Affecting the Minimum Sampling Area Determination Method: Based on Research of the Shrub Layer in Island *Pinus massoniana* Forests

**DOI:** 10.3390/biology14040372

**Published:** 2025-04-03

**Authors:** Jihong Xiao, Qingyan Wen, Zhifei Zhong, Yanqiu Xie, Yingxue Wang, Xing Cai, Yuchen Lin, Feifan Weng, Guochang Ding, Chuanyuan Deng

**Affiliations:** 1 College of Landscape Architecture and Art, Fujian Agriculture and Forestry University, Fuzhou 350002, China; xiaojihong1998@163.com (J.X.); 18379890502@163.com (Q.W.); 13170841831@163.com (Z.Z.); yanqiuxiefafu@163.com (Y.X.); wangyingxue1019@163.com (Y.W.); linycc824@163.com (Y.L.); 22319075008@fafu.edu.cn (F.W.); 2 College of Forestry, Fujian Agriculture and Forestry University, Fuzhou 350002, China; caixing1214@126.com

**Keywords:** inflection point method, precision method, Sandu Island, species–area relationship

## Abstract

Determining the minimum sampling area is crucial for accurate and cost-effective biodiversity surveys. This study identifies the main factors (e.g., species richness and evenness) influencing the inflection point method and precision method, aiming to scientifically establish the minimum sampling area for studying the shrub layer diversity of the *Pinus massoniana* community on Sandu Island, Ningde. Using 64 nested samples (20 m × 20 m) from eight sampling plots, we analyzed the species–area relationship and minimum sampling area. Key findings include the following: (1) The power function model (*S* = 4.053*A*^0.404^) best described the species–area relationship. (2) Species richness significantly influenced the inflection point method, with rare species increasing the required sampling area. (3) Species distribution uniformity and sampling methods affected model outcomes. (4) Repeated sampling from the four corners of the plots reduced bias caused by uneven species distribution. (5) Considering cost, accuracy, and inflection points, a minimum sampling area of 142 m^2^ achieved 70% sampling accuracy. This study provides a methodological framework for accurate minimum sampling area analysis and offers valuable insights for biodiversity studies of *P. massoniana* shrub layers in island ecosystems, with implications for international research on similar forest communities.

## Simple Summary

What factors influence minimum sampling area size? We addressed this question through investigations of shrub layers in island *Pinus massoniana* forests. The research shows:, the power function model was identified as the best fit for the species-area relationship. Species richness and species distribution evenness were found to be the main factors affecting the determination of the minimum sampling area. Repeated sampling from four corners of the plot proved advantageous. Considering cost and accuracy, a 142 m^2^ minimum sampling area could achieve 70% sampling accuracy. The research provides a method for analyzing the minimum sampling area and valuable insights for biodiversity studies in island ecosystems and similar forest communities worldwide.

## 1. Introduction

As the sampling area increases, the number of species within the sample plot also increases. Initially, the increase is rapid, but it gradually slows down, forming a curve known as the species–area curve [1]. The main purpose of studying the species–area relationship is to determine the minimum sampling area of a community, which refers to the smallest area that can reflect the species composition and structural characteristics of the community [2]. Confirming the minimum sampling area can reduce expensive survey costs, which is of great significance for ecological research.

The determination of the minimum sampling area is influenced by various factors, with species richness being a key consideration. Previous studies have highlighted that species richness can significantly impact the results of the minimum sampling area determination. For instance, some researchers argue that higher species richness may not always lead to an increase in the minimum sampling area, especially when comparing communities with similar species richness levels [3,4]. This raises the following question: (1) How does species richness specifically affect the minimum sampling area? Methods for determining the minimum sampling area can generally be categorized into two approaches: the precision method and the inflection point method. The precision method typically defines the minimum area based on the proportion of species within a community. For example, some studies use a threshold where a specific percentage (e.g., 70.4% [5]) of species with an occurrence frequency greater than 1 is considered sufficient for sampling precision. Similarly, the Braun-Blanquet School often adopts a standard where the minimum area includes 80% of the total species in the community [2]. Other researchers have proposed that the minimum area should encompass more than 90% of the community species [2]. In contrast, the inflection point method relies on the slope of the species–area curve to determine the minimum area. For instance, some studies identify the minimum sampling area as the point where the slope of the curve stabilizes [6], while others define it as the inflection point where the slope increases by 10% and the number of species increases by no more than 5% [7]. This leads to the second question: (2) What are the differences between the minimum sampling areas obtained by the precision method and the inflection point method? Given the variability in methods and standards, it is essential to identify the main factors influencing the minimum sampling area determined by these two approaches. This raises the third question: (3) What are the primary factors affecting the minimum sampling area obtained by the precision method and the inflection point method?

Due to their unique geographical location, islands are typically considered fragile ecosystems. Many islands have employed strategies like afforestation to mitigate ecological vulnerability [8]. However, plantations on islands often consist of monocultures, leading to simple community structures with limited resistance to pests, diseases, and natural disasters and a relative lack of understory vegetation, other ground cover plants, and microorganisms [8]. *P. massoniana*, known for its strong adaptability and drought tolerance, serves as an important species for restoring degraded lands [9]. In the Sandu Gulf area of Ningde, *P. massoniana* forests, especially on local islands, are widely distributed, occupying the largest forest area and representing the most characteristic forest type with irreplaceable ecological value [10]. These forests are semi-natural communities with a rich understory species composition [10]. However, the herb layer of local *P. massoniana* communities is significantly affected by the allelopathic effects of *Dicranopteris pedata*, resulting in very few species, which makes the shrub layer the most stable stratum within the community [10,11,12]. Shrub layer plants play a crucial role in maintaining the ecological balance and stability of the local ecosystem, underscoring their significant ecological importance [10,11,12]. Therefore, conducting research on the shrub layer of semi-natural *P. massoniana* forests, which are relatively species-rich, can help address the shortcomings of understory plant scarcity and monotonous community structure in island plantations [8]. Shrub plants serve as a crucial link between the tree layer and the herb layer, playing significant roles in substitution and connection [13]. Numerous studies have shown that the minimum sampling area varies significantly across different communities, and the shrub and herb layers are affected by variations in tree layer characteristics. Consequently, researchers often divide a community into three layers—tree, shrub, and herb (or more)—for separate analysis [14,15,16,17].

In this study, we focus on the shrub layer species of *P. massoniana* communities, utilizing the nested plot method suggested by Liu et al. [18] for the species–area curve survey. We apply three commonly used fitting models—logarithmic, power, and logistic functions—to fit the species–area curves [19,20,21,22,23]. The Akaike Information Criterion (AIC) is used to test the goodness-of-fit [24] to identify the optimal equation. We explore differences in the minimum sampling area determined by the optimal equation using the precision and inflection point methods and analyze the main factors influencing the results of these methods. This approach aims to address scientific questions (1), (2), and (3) and to scientifically establish the minimum sampling area for studying the diversity of the shrub layer in *P. massoniana* communities on Sandu Island, Ningde, improve the accuracy of research results, and reduce survey costs, providing a reference for future related studies on minimum sampling area.

## 2. Materials and Methods

### 2.1. Research Area Overview

The Sandu Gulf is located in the southeast of Ningde City, Fujian Province, at the midpoint of China’s 18,400 km “Golden Coastline”, about 30 km from the Ningde urban area. It is a world-class natural deep-water harbor. The area comprises 126 islands, 17 of which are inhabited. The largest is Sandu Island, covering approximately 27.74 km^2^ and serving as the seat of the Sandu Town government. The study area is characterized by a typical subtropical maritime monsoon climate with hilly terrain. The primary soil types are red soil and yellow soil. The island is predominantly covered by secondary *P. massoniana* coniferous and broad-leaved mixed forest [10].

### 2.2. Community Survey

**Figure 1:**
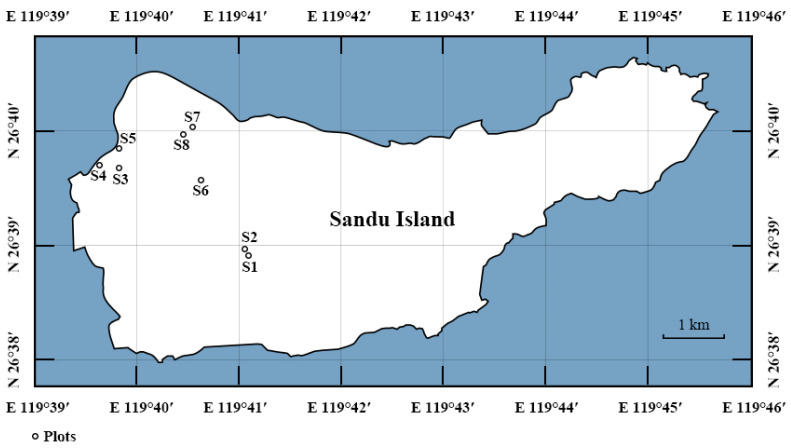
Distribution map of sampling plots.

**Figure 2:**
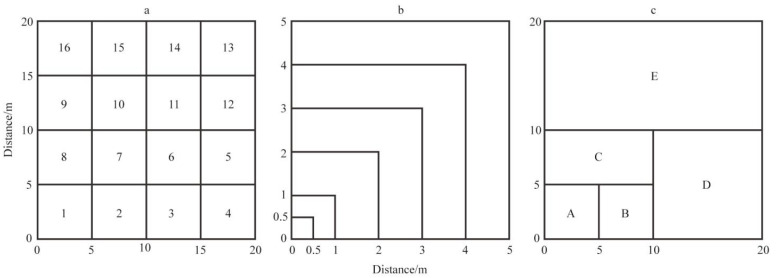
Schematic design of the sample plot.

Based on preliminary field inspections, a typical plot method was employed from June to July 2022 to establish sample plots on Sandu Island. Eight forest plots, each 20 m × 20 m, were set up with *P. massoniana* as the dominant species in the tree layer (Figure 1). The basic information for the plots is provided in Appendix A. The selection criteria for the plots included consideration of varying slopes, positions, and elevations, minimal disturbance, moderate community canopy density, a buffer zone of over 30 m, and representativeness of the community. Additionally, the selected plots were mature stands protected by local policies, with no human management for many years, ensuring they represented semi-natural communities. Following the study by Ren et al. [25], an adjacent grid method was used to divide each 20 m × 20 m plot into sixteen 5 m × 5 m subplots (Figure 2a). Each plot’s first, fourth, thirteenth, and sixteenth subplots were further subdivided into smaller plots of 0.5 m × 0.5 m, 1 m × 1 m, 2 m × 2 m, 3 m × 3 m, 4 m × 4 m, and 5 m × 5 m (Figure 2b). Using a nested sampling method, the 5 m × 5 m subplots were then combined into plots of 5 m × 10 m, 10 m × 10 m, 10 m × 20 m, and 20 m × 20 m (Figure 2c). This configuration resulted in 10 area gradients ranging from a minimum of 0.25 m^2^ to a maximum of 400 m^2^. The species present in the shrub layer were recorded for the 16 5 m × 5 m subplots and the 6 area gradients within subplots 1, 4, 13, and 16. To minimize interference from species distribution variations, area expansion began from the four corners, specifically from the 0.25 m^2^ of subplots 1, 4, 13, and 16. Each corner was expanded to 25 m^2^ and 100 m^2^, with datasets being further expanded in two consistent directions, resulting in eight groups of different plot data per site and a total of 64 groups of 20 m × 20 m plot data. Specific data can be found in Appendix B.

### 2.3. Species–Area Relationship Models and Minimum Sampling Area Equations

Three commonly used species–area relationship models—logarithmic, power function, and logistic models [19,20,21,22,23]—were selected for functional model fitting and related calculations in nested sampling methods (Table 1).

### 2.4. Evaluation of Fit for Species–Area Relationship Models and Determination of Minimum Sampling Area

The least squares method was used to test the logarithmic model, while the Gauss–Newton algorithm was applied to test the power function and logistic models [20]. The value of *R*^2^ alone cannot accurately determine the goodness-of-fit; further testing is required [26]. Therefore, the goodness-of-fit for the three models was evaluated using the Akaike Information Criterion (AIC) [24]. A smaller AIC value indicates a better model fit and more accurate predictions.

In this study, two methods, namely the precision method and the inflection point method, were used to analyze the minimum sampling area. Based on the optimal model, seven different estimation accuracies (50%, 60%, 70%, 75%, 80%, 85%, 90%, and 95%) were selected to calculate the minimum sampling area [26,27]. At the same time, the inflection point method was used to calculate the minimum sampling area, and the slope of the curve of <0.1 was taken as the standard for identifying the inflection point [28,29], upon which the minimum sampling area was determined. The specific calculation steps of the inflection point are as follows: the first derivative of the optimal fitting model was calculated, the minimum sampling area *A* with different precision values was approximated to the first derivative, and the slope of the curve corresponding to the area was then used to find the inflection point.

For data analysis in this study, the c value in the logistic function was obtained by using Mathematica 12.2 software [30], and the AIC value was calculated by using “MuMIn” package in R 4.1.2 software program; all other data analysis and mapping were performed by using SPSS 26, Excel 2019, etc.

## 3. Results

### 3.1. Species–Area Relationship in the Shrub Layer

The mean values from 8 quadrats across 8 sampling plots on Sandu Island, totaling 64 quadrats, alongside 9 groups of field survey data (Table 1), were used for curve-fitting using functions (1) to (3). Each parameter for the related species–area curve equations was obtained (Table 2). According to *R*^2^ values greater than 0.8 and *p*-values of 0.000, the species–area relationships for the three target plants across three types of island forest communities aligned with the three fitting functional models. Among the nine data groups, the power function exhibited the highest *R*^2^. The goodness-of-fit was further assessed using the Akaike Information Criterion (AIC), identifying the best species–area curve from the nine data groups. The power function model had the lowest AIC value, indicating the best fit, followed by the logistic function model, while the logarithmic model had the poorest fit. These results were consistent with the *R*^2^ findings. Consequently, the optimal fitting models for the species–area relationship among the nine data groups were all power function models, expressed as: *S*_S1_ = 4.976*A*^0.348^, *S*_S2_ = 2.438*A*^0.412^, *S*_S3_ = 4.353*A*^0.448^, *S*_S4_ = 4.352*A*^0.411^, *S*_S5_ = 3.965*A*^0.401^, *S*_S6_ = 3.956*A*^0.428^, *S*_S7_ = 4.195*A*^0.415^, *S*_S8_ = 4.043*A*^0.37^, and *S*_All_ = 4.053*A*^0.404^.

**Figure 3:**
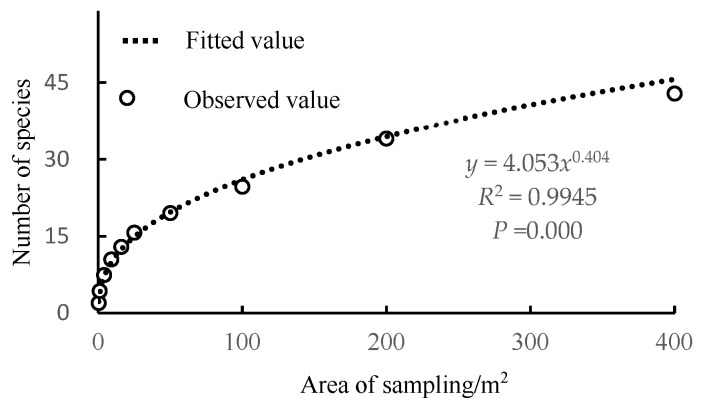
The best fitting results of the species–area curve of shrub layer.

The overall mean value of the dataset was fitted using the optimal power function model curve (Figure 3). As shown in Figure 3 and Appendix B, at a sampling area of 200 m^2^, the average number of species was 34.09, constituting 79.5% of the total species count. When the sampling area increased to 400 m^2^, the average number of species rose to 42.88, only 20.5% of the total species. This indicates that, as the sampling area expands, the number of plant species in the sample also increases, initially rapidly and then more gradually. The fitted values closely matched the observed values, indicating a successful curve-fitting process. Thus, the species–area curve for the shrub layer in the *P. massoniana* community on Sandu Island was determined as *S* = 4.053*A*^0.404^.

### 3.2. Minimum Sampling Area in the Shrub Layer

The equation for the minimum sampling area (5) based on the power function model (2) with the best goodness-of-fit test was selected to determine the minimum sampling area for the species diversity survey (Table 1). The equations for the minimum sampling area of nine groups of data are as follows: *A*_S1_ = (38*ρ*/4.976)^1/0.348^, *A*_S2_ = (27*ρ*/2.438)^1/0.412^, *A*_S3_ = (52*ρ*/4.353)^1/0.448^, *A*_S4_ = (52*ρ*/4.352)^1/0.411^, *A*_S5_ = (41*ρ*/3.965)^1/0.401^, *A*_S6_ = (50*ρ*/3.956)^1/0.428^, *A*_S7_ = (42*ρ*/4.195)^1/0.415^, *A*_S8_ = (41*ρ*/4.043)^1/0.37^, and *A*_All_ = (42.88*ρ*/4.053)^1/0.404^.

Seven different estimation accuracies of 50%, 60%, 70%, 75%, 80%, 85%, 90%, and 95% were selected to determine the minimum sampling area of nine groups of data (Table 3). The results showed that, with the increase in the estimation accuracy, the minimum sampling area of nine groups of shrub layer data for the species diversity survey gradually expanded, and the corresponding slope of the species–area curve gradually decreased. According to the inflection point method, to ensure accuracy within the ranges of 50% to 95%, when the accuracy of the sampling plot S1 was within the range of 60% to 70%, the slope of the curve changed from 0.1 to 0.08, while when the accuracy was 70%, the slope of the curve was 0.08 < 0.1, and the minimum sampling area for shrub survey in the sampling plot S1 was 79.4 m^2^~123.6 m^2^. When the sampling accuracy was 50%, the curve slope was steep, and the minimum sampling area was 47 m^2^, which is inconsistent with the actual situation. Similarly, when the accuracy ranged from 50% to 95%, the minimum sampling area or the sampling interval of S2~S8 and all sampling plots had the values of 63.7 m^2^, 200.6 m^2^~226.3 m^2^, 120.6 m^2^~175.5 m^2^, 94.8 m^2^~139.2 m^2^, 113.7 m^2^~163 m^2^, 128.8 m^2^~150.4 m^2^, 80.5 m^2^, and 97 m^2^~142 m^2^, respectively.

As presented in Table 4, using only the inflection point method without considering accuracy—specifically when the slope of the fitting curve was less than 0.1—the minimum sampling areas for plots S1 to S8, and the overall plots, were 79.4 m^2^, 50.6 m^2^, 217.4 m^2^, 133.9 m^2^, 101.4 m^2^, 140.7 m^2^, 132.2 m^2^, 73.3 m^2^, and 109 m^2^, respectively. During this analysis, the estimated number of species according to the fitting curve closely matched the actual observed species count, indicating a high degree of curve-fitting accuracy and more precise determination of the minimum sampling area.

**Figure 4:**
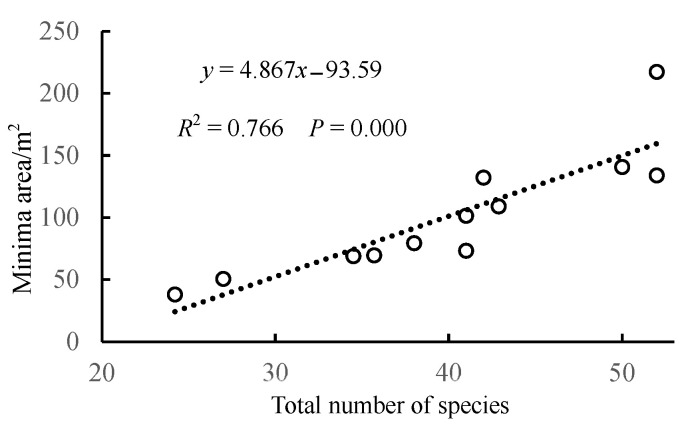
Regression analysis between the total number of species in the plots and the minimum area obtained by the inflection point method.

Using the data from this study (Table 4) and the research on the minimum sampling area of the shrub layer in *Acacia confusa*, *Eucalyptus citriodora*, and *Celtis sinensis* communities on Langqi Island, Fujian, China [24], which employed the same plot setup and sampling methods as this study, a regression analysis was conducted between the minimum sampling area obtained by the inflection point method and the total number of species in the plots. The results are shown in Figure 4. The results indicate a highly significant positive correlation (*p* < 0.001) between the total number of species in the plots and the minimum area obtained by the inflection point method.

## 4. Discussion

Many scholars have noted that species–area curves are dependent on plot scale, with medium-scale plots fitting the power function model more accurately [31]. In this study, the fitting results for all plots showed that the power function was optimal, likely because the plot sizes were of medium scale [26]. As illustrated in Figure 3, the power function curve initially increases rapidly and then slows down, with the corresponding curve slope starting large and then gradually decreasing. The minimum sampling area analysis at different precision levels (Table 3) indicates that, as estimation accuracy increases, the required sampling area also increases, and the slope of the species–area curve gradually decreases. These results align with the typical behavior of species–area curves: as the sampling area expands, the number of species increases rapidly at first, then more slowly [32,33]. This suggests that the power function model indeed fits well at medium scales.

Keeley [3] suggested that different species abundance distributions might lead to varying species–area curve outcomes, subsequently affecting the determination of minimum sampling area. Xiao et al. [26] argued that species–area curves, which only account for species richness, may overemphasize the role of rare species, leading to an increase in the calculated minimum sampling area. This study, through an analysis of shrub layer plants in different island communities—*P. massoniana* (Table 4), *A. confusa*, *E. citriodora*, and *C. sinensis* [26]—using the same sampling method, shows (Table 4 and Figure 4) that the minimum sampling area increases with the total number of species in a plot, indicating a positive correlation (*p* < 0.001) between these variables. It is evident that species richness influences the minimum sampling area across different study regions and communities. Thus, when calculating the minimum area, including rare species increases the species count and thus the minimum sampling area determined by the inflection point method. Conversely, excluding rare species reduces the minimum sampling area. However, this is not absolute. For instance, plot S6 (50 species) has a larger minimum sampling area than plot S4 (52 species), and plot S3 (52 species) has a larger area than S4 (52 species), mainly due to uneven species distribution within the plots [4,34]. From Appendix B, it is evident that plots S3 and S6 exhibit higher species counts at sampling areas of 200 m^2^, 100 m^2^, and 50 m^2^ compared to plot S4, resulting in a larger opening of their power function fitting curves relative to the x-axis, thereby increasing the slope and the minimum area determined by the inflection point method [21]. In contrast, minimum areas determined by accuracy criteria differ. At the same precision levels (75–95%), plots S3 and S7 show the smallest minimum sampling areas. This is because, at sampling areas of 100 m^2^ to 200 m^2^, these plots already encompass a large proportion of the total species count, resulting in a smaller growth rate in species count when the area doubles to 400 m^2^. The accuracy method calculates minimum sampling area based on species proportion [2]; thus, under identical precision conditions, plots S3 and S7 have relatively smaller minimum areas, with similar conclusions applicable to other sampling areas and precision levels. It is worth mentioning that, in our previous research [11], we found that the species richness of the shrub layer was significantly influenced by soil pH, organic matter, and wind speed of the *Pinus massoniana* community on Sandu Island. The higher the soil pH and wind speed, the greater the species richness in the island community. On islands, soil stability primarily depends on soil organic matter [11]. A lack of soil organic matter can impair soil nutrients, disrupt island habitats, and reduce species richness, ultimately destabilizing the community. Additionally, factors such as distance from the coastline and slope aspect can influence the levels of pH and organic matter, thereby leading to variations in species richness [10]. Therefore, when setting up the sample plots in this study, these factors were taken into account, resulting in significant differences in the total number of species among different plots (Table 4).

Appendix B also indicates that, within the same plot, using a nested sampling method from different sampling points results in varying species counts for the same sampling area. This is particularly evident in plots with uneven species distribution [4], such as plot S5, where the species count variance at 25 m^2^ reached 13. This finding aligns with the results of large-scale studies by He et al. [35], who analyzed plots ranging from 25 to 60 ha by varying sampling directions and found that topographic heterogeneity and species variability significantly influence the outcomes of minimum sampling area determinations. Therefore, when conducting species–area relationship studies using the nested sampling method, it is insufficient to randomly sample from just one corner of the plot. Instead, repeated sampling from all four corners is recommended to effectively minimize bias caused by uneven species distribution.

From the accuracy method perspective, the minimum sampling areas required for precisions of 50%, 60%, 70%, 75%, 80%, 85%, 90%, and 95% are 61.8 m^2^, 97 m^2^, 142 m^2^, 168.5 m^2^, 197.7 m^2^, 229.7 m^2^, 264.6 m^2^, and 302.5 m^2^, respectively, corresponding to 3, 4, 6, 7, 8, 10, 11, and 13 shrub plots of 5 m × 5 m each. According to the inflection point method alone, the minimum sampling area is 109 m^2^ or five shrub plots of 5 m × 5 m. By combining both the accuracy and inflection point methods, the optimal minimum sampling area should be set at 142 m^2^, equivalent to six shrub plots of 5 m × 5 m, achieving 70% sampling accuracy. Given the study area is an inland island with richer species diversity than offshore islands, this minimum area can be widely applied to *P. massoniana* forests on islands.

## 5. Conclusions

This study highlights the importance of species richness, distribution uniformity, and sampling methods in determining the minimum sampling area for the shrub layer of the *P. massoniana* community on Sandu Island. The power function model (*S* = 4.053*A*^0.404^) was identified as the optimal model for medium-scale species–area relationships, while repeated sampling from the four corners of plots effectively minimized bias. For future research, extending the analysis to smaller and larger scales is recommended to compare differences in species–area relationships. Additionally, while current studies on shrub layer plants in inland forest communities often use 5 m × 5 m plots, larger-scale sampling should be adopted in regions with high biodiversity to ensure comprehensive and accurate assessments. These findings provide a methodological framework for biodiversity studies in island ecosystems and contribute to international research on similar forest communities.

## Figures and Tables

**Table 1:** Commonly used species–area relationship fitting models in nested sampling method.

Function	Species–Area Fitting Function Model	Minimum Sampling Area Equation
Logarithm	(1) *S* = *a* + *b*ln*A*	(4) *A* = exp[(*ρS_t_* − *a*)/*b*]
Power	(2) *S* = *aA^b^*	(5) *A* = (*ρS_t_*/*a*)^1/*b*^
Logistic	(3) *S* = *c*/(1 + *a*e^−*bA*^)	(6) *A* = −{ln[(*c*/*ρS_t_* − 1)/*a*]}/*b*

*A* represents the sample ground area; *S* represents the number of species in the corresponding sample ground; *a*, *b*, *c* are the parameters to be estimated; *S_t_* represents the total number of species in the surveyed community; *ρ* represents the ratio of the expected species to the total species; The same below.

**Table 2:** Fitting results of species–area relationship.

Plot No.	Function	Parameter			*R* ^2^	*p*	AIC	Plot No.	Function	Parameter			*R* ^2^	*p*	AIC
		a	b	c						a	b	c			
S1	(1)	4.314	4.478	-	0.901	0.000	58.14	S6	(1)	1.810	6.154	-	0.827	0.000	70.96
	(2)	4.976	0.348	-	0.982	0.000	−10.66		(2)	3.956	0.428	-	0.997	0.000	−23.78
	(3)	0.112	0.981	38.04	0.927	0.000	26.41		(3)	0.134	0.980	50.10	0.944	0.000	23.84
S2	(1)	1.791	3.205	-	0.863	0.000	55.08	S7	(1)	3.256	5.526	-	0.913	0.000	60.87
	(2)	2.438	0.412	-	0.973	0.000	−3.41		(2)	4.195	0.415	-	0.985	0.000	−9.50
	(3)	0.239	0.978	27.01	0.917	0.000	30.70		(3)	0.127	0.977	42.01	0.931	0.000	29.66
S3	(1)	3.098	6.810	-	0.899	0.000	66.79	S8	(1)	2.144	4.775	-	0.804	0.000	67.39
	(2)	4.353	0.448	-	0.981	0.000	−5.30		(2)	4.043	0.370	-	0.993	0.000	−19.07
	(3)	0.122	0.976	52.01	0.931	0.000	30.41		(3)	0.148	0.981	41.10	0.955	0.000	20.37
S4	(1)	2.291	6.129	-	0.825	0.000	70.97	All	(1)	2.670	5.268	-	0.867	0.000	64.68
	(2)	4.352	0.411	-	0.996	0.000	−22.83		(2)	4.053	0.404	-	0.994	0.000	−19.81
	(3)	0.133	0.980	52.06	0.935	0.000	26.14		(3)	0.141	0.978	42.90	0.937	0.000	23.12
S5	(1)	2.657	5.071	-	0.863	0.000	64.26								
	(2)	3.965	0.401	-	0.992	0.000	−15.93								
	(3)	0.145	0.978	41.02	0.942	0.000	26.56								

S: Sandu Island; AIC: Akaike Information Criterion; All: data consisting of the mean values of sample plots S1~S8; the same below.

**Table 3:** Area sampled for species diversity surveys in shrub layer with different precision.

Plot No.	Different Estimation Precision															
	*ρ* = 0.5		*ρ* = 0.6		*ρ* = 0.7		*ρ* = 0.75		*ρ* = 0.8		*ρ* = 0.85		*ρ* = 0.9		*ρ* = 0.95	
	Area/ m^2^	Slope	Area/ m^2^	Slope	Area/ m^2^	Slope	Area/ m^2^	Slope	Area/ m^2^	Slope	Area/ m^2^	Slope	Area/ m^2^	Slope	Area/ m^2^	Slope
S1	47.0	0.141	79.4	0.100	123.6	0.075	150.7	0.066	181.4	0.058	215.9	0.052	254.4	0.047	297.2	0.042
S2	63.7	0.087	99.2	0.067	144.1	0.054	170.4	0.049	199.3	0.045	230.9	0.041	265.3	0.038	302.5	0.035
S3	54.0	0.216	81.2	0.172	114.5	0.142	133.5	0.131	154.2	0.121	176.6	0.112	200.6	0.104	226.3	0.098
S4	77.4	0.138	120.6	0.106	175.5	0.085	207.6	0.077	242.9	0.070	281.5	0.065	323.5	0.059	369.0	0.055
S5	60.2	0.137	94.8	0.104	139.2	0.083	165.4	0.075	194.2	0.068	226.0	0.062	260.6	0.057	298.2	0.052
S6	74.3	0.144	113.7	0.112	163.0	0.092	191.5	0.084	222.7	0.077	256.6	0.071	293.2	0.066	332.7	0.061
S7	48.5	0.180	75.2	0.139	109.1	0.112	128.8	0.102	150.4	0.093	174.1	0.085	199.8	0.079	227.6	0.073
S8	80.5	0.094	131.7	0.069	199.7	0.053	240.7	0.047	286.6	0.042	337.6	0.038	394.0	0.035	456.0	0.032
All	61.8	0.140	97.0	0.107	142.0	0.085	168.5	0.077	197.7	0.070	229.7	0.064	264.6	0.059	302.5	0.054

**Table 4:** Information at the inflection point.

Plot No.	Minima Area	Number of Estimates Species	Number of Actual Species	Total Number of Species
S1	79.4	22.8	18.25~22.75	38.00
S2	50.6	12.3	12.00~14.13	27.00
S3	217.4	48.5	43.50~52.00	52.00
S4	133.9	32.6	26.75~38.25	52.00
S5	101.4	25.3	22.75~34.00	41.00
S6	140.7	32.9	26.75~40.25	50.00
S7	132.2	31.8	28.50~35.25	42.00
S8	73.3	19.8	16.63~21.25	41.00
All	109.0	27.0	24.70~34.09	42.88

## Data Availability

The original contributions presented in this study are included in the article. Further inquiries can be directed to the corresponding authors.

## Appendix A

**Table A1:** Basic information of the sample site.

Sample Site No.	Longitude	Latitude	Elevation/(m)	Slope Aspect	Slope/(°)	Slope Position	Crown Density
S1	119°40′47.66″	26°39′07.25″	42.4	SE160	30	Middle	0.75
S2	119°40′47.12″	26°39′09.55″	81.1	SE160	30	Up	0.75
S3	119°39′33.26″	26°39′53.18″	178.4	N353	30	Up	0.80
S4	119°39′20.11″	26°39′51.20″	71.9	NW312	30	Middle	0.75
S5	119°39′32.59″	26°39′57.99″	123.2	W261	37	Up	0.68
S6	119°40′22.00″	26°39′43.00″	175.5	SE155	32	Middle	0.80
S7	119°40′16.47″	26°40′10.93″	40.7	NW289	24	Low	0.75
S8	119°40′10.60″	26°40′07.14″	96.0	NE23	23	Low	0.80

SE: Southeast; N: North; NW: Northwest; W: West; NE: Northeast.

## Appendix B

**Table A2:** Data of nested samples.

Sample Site No.	Quadrat No.	Number of Species in Different Sampled Areas									
		0.25 m^2^	1 m^2^	4 m^2^	9 m^2^	16 m^2^	25 m^2^	50 m^2^	100 m^2^	200 m^2^	400 m^2^
S1	1	4	8	15	18	19	20	21	23	31	38
	2	4	8	15	18	19	20	20	23	29	38
	3	3	7	9	16	18	19	20	25	31	38
	4	3	7	9	16	18	19	21	25	30	38
	5	1	2	5	9	10	11	14	18	30	38
	6	1	2	5	9	10	11	13	18	30	38
	7	2	4	6	8	11	13	18	25	29	38
	8	2	4	6	8	11	13	19	25	30	38
	Mean	2.50	5.25	8.75	12.75	14.50	15.75	18.25	22.75	30.00	38.00
S2	1	1	3	5	7	9	9	11	13	24	27
	2	1	3	5	7	9	9	13	13	18	27
	3	2	5	7	10	13	13	16	22	24	27
	4	2	5	7	10	13	13	17	22	23	27
	5	0	2	3	4	6	8	10	11	23	27
	6	0	2	3	4	6	8	9	10	16	27
	7	1	2	3	6	9	9	10	11	18	27
	8	1	2	3	6	9	9	10	11	16	27
	Mean	1.00	3.00	4.50	6.75	9.25	9.75	12.00	14.13	20.25	27.00
S3	1	2	4	6	10	13	17	23	35	39	52
	2	2	4	6	10	13	17	14	35	44	52
	3	2	6	14	19	20	22	25	28	39	52
	4	2	6	14	19	20	22	26	28	42	52
	5	1	3	5	8	13	19	28	36	42	52
	6	1	3	5	8	13	19	27	36	49	52
	7	2	6	11	17	20	23	31	40	44	52
	8	2	6	11	17	20	23	31	40	49	52
	Mean	1.75	4.75	9.00	13.50	16.50	20.25	25.63	34.75	43.50	52.00
S4	1	1	7	9	14	15	17	21	29	37	52
	2	1	7	9	14	15	17	23	29	45	52
	3	2	4	7	10	13	17	20	24	37	52
	4	2	4	7	10	13	17	20	24	34	52
	5	4	5	7	12	13	13	17	22	34	52
	6	4	5	7	12	13	13	17	22	37	52
	7	2	3	7	11	15	18	23	32	45	52
	8	2	3	7	11	15	18	27	32	37	52
	Mean	2.25	4.75	7.50	11.75	14.00	16.25	21.00	26.75	38.25	52.00
S5	1	1	2	4	7	8	9	15	17	37	41
	2	1	2	4	7	8	9	11	17	24	41
	3	2	4	8	10	15	22	23	34	37	41
	4	2	4	8	10	15	22	31	34	38	41
	5	4	7	14	15	17	19	21	23	38	41
	6	4	7	14	15	17	19	19	23	37	41
	7	1	3	6	6	10	14	16	17	24	41
	8	1	3	6	6	10	14	14	17	37	41
	Mean	2.00	4.00	8.00	9.50	12.50	16.00	18.75	22.75	34.00	41.00
S6	1	1	2	8	13	16	22	25	29	41	50
	2	1	2	8	13	16	22	25	29	40	50
	3	3	6	8	11	12	14	20	25	36	50
	4	3	6	8	11	12	14	17	25	40	50
	5	3	6	8	11	12	14	19	24	40	50
	6	3	6	8	11	12	14	18	24	44	50
	7	1	3	6	8	9	14	20	29	40	50
	8	1	3	6	8	9	14	21	29	41	50
	Mean	2.00	4.25	7.50	10.75	12.25	16.00	20.63	26.75	40.25	50.00
S7	1	2	3	8	9	14	20	24	26	29	42
	2	2	3	8	9	14	20	23	26	35	42
	3	3	5	8	10	12	18	23	25	29	42
	4	3	5	8	10	12	18	19	25	36	42
	5	2	6	10	14	19	24	27	33	36	42
	6	2	6	10	14	19	24	27	33	41	42
	7	1	3	6	8	11	16	23	30	35	42
	8	1	3	6	8	11	16	22	30	41	42
	Mean	2.00	4.25	8.00	10.25	14.00	19.50	23.50	28.50	35.25	42.00
S8	1	1	2	4	6	8	10	12	20	28	41
	2	1	2	4	6	8	10	16	20	33	41
	3	3	4	5	8	10	11	15	17	29	41
	4	3	4	5	8	10	11	15	17	28	41
	5	2	6	7	9	12	15	18	23	29	41
	6	2	6	7	9	12	15	19	23	35	41
	7	4	6	10	12	12	13	18	25	33	41
	8	4	6	10	12	12	13	20	25	35	41
	Mean	2.50	4.50	6.50	8.75	10.50	12.25	16.63	21.25	31.25	41.00
Grand mean		2.00	4.34	7.47	10.50	12.94	15.72	19.55	24.70	34.09	42.88
